# Dissection of Larval and Pupal Wings of *Bicyclus anynana* Butterflies

**DOI:** 10.3390/mps3010005

**Published:** 2020-01-10

**Authors:** Tirtha Das Banerjee, Antónia Monteiro

**Affiliations:** 1 Department of Biological Sciences, National University of Singapore, 14 Science Drive 4, Singapore 117543, Singapore; antonia.monteiro@nus.edu.sg; 2 Yale-NUS College, 10 College Avenue West, Singapore 138609, Singapore

**Keywords:** wing dissection, *Bicyclus anynana*, butterflies

## Abstract

The colorful wings of butterflies are emerging as model systems for evolutionary and developmental studies. Some of these studies focus on localizing gene transcripts and proteins in wings at the larval and pupal stages using techniques such as immunostaining and in situ hybridization. Other studies quantify mRNA expression levels or identify regions of open chromatin that are bound by proteins at different stages of wing development. All these techniques require dissection of the wings from the animal but a detailed video protocol describing this procedure has not been available until now. Here, we present a written and accompanying video protocol where we describe the tools and the method we use to remove the larval and pupal wings of the African Squinting Bush Brown butterfly *Bicyclus anynana*. This protocol should be easy to adapt to other species.

## 1. Introduction

Multiple studies on butterflies are focused on understanding the evolutionary and developmental genetics of their colorful wing patterns. Labs around the world have worked with different species to address a variety of questions at the intersection of these fields. Examples include the discovery of the involvement of the gene *optix* in wing pattern mimicry of *Heliconius* butterflies [1,2]; discovery of *doublesex* as a mimicry supergene in *Papilio polytes* [3]; involvement of Wnt signaling in wing pattern of butterflies such as *Junonia coenia*, *Heliconius erato* and *Vanesssa cardui* [4,5,6]; involvement of the genes *spalt* and *BarH-1* in the wing pigmentation of multiple *Pieris* and *Colias* species [7,8,9]; and the involvement of calcium signaling in wing patterning in *Junonia orithya* [10] to mention a few. 

*Bicyclus anynana* has been a popular system to study wing patterning, especially the eyespots, which are novel traits to the nymphalid lineage [11]. Many of the studies have been focused on identifying the local expression of proteins such as Spalt [12], Engrailed/Invected [12], Distal-less [13], Antennapedia [14,15], Notch [16], Cubitus-interruptus [17], Ecdysone Receptor [18], Ultrabithorax [15,17], and the expression of gene transcripts such as *hedgehog* [14], *apterous* [19], *patched* [16], *wingless* [20], *doublesex* [21] and *decapentaplegic* [22] in the developing wing. Gene expression and RNAi studies have proposed that many wound healing network genes are expressed in eyespots [23], that a positional-information mechanism is involved in the formation of the concentric rings [16,20], and that a reaction-diffusion mechanism is involved in setting up the eyespot centers [22] using gene expression and functional analysis via CRISPR-Cas9 [24,25]. All these studies require the removal of wings from the bodies of larvae and/or pupae to examine patterns of gene expression. Protocols describing the dissection of larval and pupal wings have been previously published [26,27]. However, the protocols are brief and have no accompanying video, making it difficult for newcomers in the field to follow. 

In this paper, we describe the process of larval and pupal wing removal using a video and explain the process along with all the tools and chemicals needed in the main text below. This protocol can also supplement other similar experiments such as live cell imaging in vivo used to understand cell differentiation and dynamics [28,29].

## 2. Experimental Design

### 2.1. Required Materials and Equipment

#### 2.1.1. Materials

- Curved tweezers (Dumont; Dumont Switzerland, Montignez, Switzerland; Cat. No.: 11274-20);
- Fine straight tweezers (Dumont; Dumont Switzerland; Cat. No.: 11254-20);
- Flat spatula (Thomas Scientific; Thomas Scientific, Swedesboro, NJ, USA; Cat. No.: 1208Y75);
- Regular straight tweezers (Dumont; Dumont Switzerland; Cat. No.: 0203-5-PO);
- Superfine Vannas scissors 8 cm (World Precision Instruments; World Precision Instruments, Sarasota, FL, USA; Cat. No.: 501778);
- Blade holder (Swann-Morton No. 4; Swann-Morton, Sheffield, UK; Cat. No.: 0934);
- Blades (Swann-Morton No. 4; Swann-Morton, Sheffield, UK; Cat. No.: 0115);
- Glass spot plate (PYREX^TM^; Corning, Corning, NY, USA; Cat. No.: 722085);
- Dissection silicone plate (Dragon Skin 30 Mould Making Silicone Rubber; Cat. No.: 0751635278417. Petri plate; Sigma-Aldrich; Sigma-Aldrich, Singapore; Cat. No.: P5981-100EA);
- Insect pins (BioQuip; BioQuip, Rancho Dominguez, CA, USA; Cat. No.: 1208B2).

#### 2.1.2. Equipment

- Zeiss Dissection Microscope (Carl-Zeiss, Jena, Germany; Stemi 305)

#### 2.1.3. Reagents

- NaCl (Sigma-Aldrich; Sigma-Aldrich, Singapore; Cat. No.: S9888-500G);
- K_2_HPO_4_ (Sigma-Aldrich; Sigma-Aldrich, Singapore; Cat. No.: P3786-500G);
- KH_2_PO_4_ (Sigma-Aldrich; Sigma-Aldrich, Singapore; Cat. No.: 229806-250G);
- RNAseZap (Themo Fisher Scientific; Thermo Fisher Scientific, Waltham, MA, USA; Cat. No.: AM9780).

## 3. Procedure

### 3.1. Preparation for Dissection

1. Transfer 500 µL of 1 × PBS into each well of the spot glass plate.
2. Transfer around 100 mL of 1 × PBS into the dissection well plate.
3. Wash the dissection tools in 70% ethanol prior to dissection.
4. Freeze anaesthetize the larvae and pupae on ice for 10–20 min.
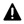
**CRITICAL STEP**: If you are performing experiments involving RNA, it is recommended that all the equipment is wiped with RNAseZap.

### 3.2. Dissection of Larval Wings

**Figure 1:**
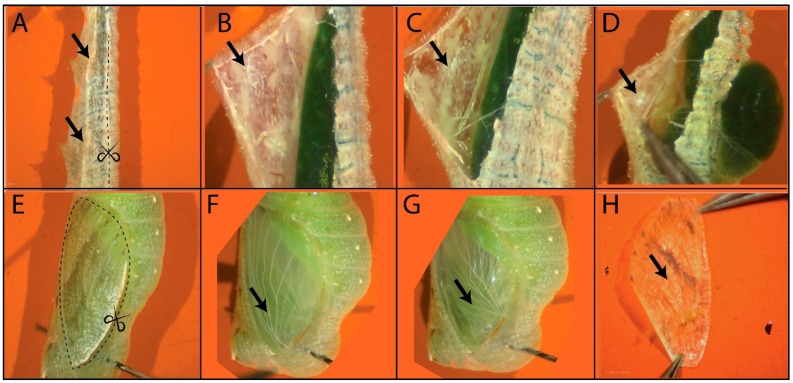
Dissection of larval and pupal wings of *Bicyclus anynana*. (**A**). Larval wings are located laterally (black arrows), dorsal to the second and third thoracic legs. The region for incision is marked by a dotted line. (**B**). A larval forewing is located dorsally to the second thoracic leg. (**C**). A larval hindwing is located beside the third thoracic leg. (**D**). Early larval wings can be identified by finding the white tissue (black arrow) around the thoracic legs. Furthermore, to release the pressure due to the gut it is recommended to make an initial dorsal incision (through which the gut can extend) before the lateral incision is made. (**E**). For the dissection of pupal wings make incision as marked by the dotted line. (**F**). Pupal forewing. (**G**). Pupal hindwing. (**H**). Early (16–26 h after pupation) pupal forewing.

1. Pick one larva from the ice and carefully secure it in the dissection plate with the help of two pins. One pin should be placed immediately posterior to the head capsule, and the second pin at the end of the abdomen. It is recommended to stretch the larva, before placing the second pin, to make the dissection and removal of wings easier.
2. The wings are located around the second and third thoracic legs.
3. Hold the epidermis of the larva using a straight tweezer and using the Vannas scissors make an incision, as indicated in Figure 1A.
4. After the incision try to find the hindwing around the third thoracic leg (Figure 1C). If you are working with a young fifth instar larva, the wing can be identified adjacent to a white lump of tissue around the thoracic leg (Figure 1D). This white tissue at the base of the wing will become the trachea that will invade the wing blade and is the preferred spot for handling the wing to avoid touching the actual wing tissue attached.
5. Make cuts to the tissues/trachea attached on both sides of the wing and carefully pull out the wing (touching only the white tissue) using a fine tweezer.
6. Transfer the hindwing to one of the wells of the glass plate.
7. The forewing is present just a few millimeters above the third thoracic leg (Figure 1A). Perform the cuts as with the hindwing and pull out the wing using a fine tweezer.
8. Transfer the forewing to one of the wells of the glass plate.
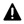
**CRITICAL STEP:** Be careful not to touch the wing membrane as even a gentle contact with the tweezer can damage the wing.

### 3.3. Dissection of Pupal Wings

1. Secure a pupa in the dissection plate with the help of two fine pins.
2. Make incisions using a fine blade at the region marked in Figure 1E.
3. Remove the cuticle using a curved tweezer. The forewing should be visible at the surface of the pupa (Figure 1F). If you are working with a wing that is less than 26 h old, the forewing might be still attached to the cuticle (Figure 1H). Using a straight tweezer on one hand, hold the cuticle down and using a curved tweezer on the other hand, gently dislodge the wing from the cuticle, scraping the wing from underneath in gentle nudges, and finally pull out the wing.
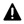
**CRITICAL STEP:** Make sure that the forewing is free from any attachment to the cuticle.
4. After the forewing is free, transfer the wing to one of the wells of the glass plate using a flat spatula. Hold the wing by the hinge region, and do not touch the rest of the wing blade with the tweezers. Hold the wing with the tweezers against the spatula until the spatula breaks the liquid-air surface interface. You can also use the tweezers to gently help slide the wing into the glass wells.
5. To remove the hindwing (Figure 1G), make an incision around the wing using a fine blade and carefully pull out a glassy (peripodial) membrane on top of the hindwing.
6. After the membrane is removed, make a cut at the wing-hinge region and pull out the wing using a curved tweezer.
7. After the hindwing is free, transfer the wing to one of the wells of the glass plate using a flat spatula.
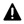
**CRITICAL STEP:** Be careful not to touch the wing membrane as even a gentle contact with the tweezer can damage the wing.

## 4. Expected Results

### Larval Wings and Pupal Wing

**Figure 2:**
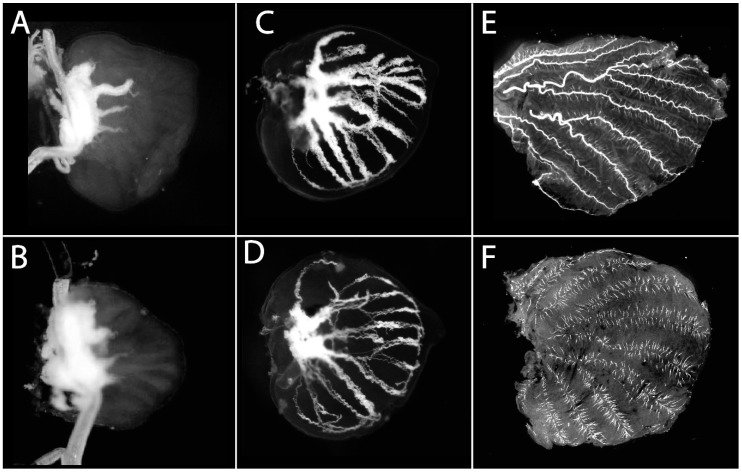
Larval and Pupal wings of *Bicyclus anynana*. (**A**) Early larval forewing showing the prominent white tissue that will differentiate into the trachea. Wings should be handled in this region during dissections; (**B**) Early larval hindwing; (**C**) Late larval forewing; (**D**) Late larval hindwing; (**E**) Pupal forewing; (**F**) Pupal hindwing.

Larval wings at an early developmental stage are marked by a lack of tracheal invasion in the wing disc and a prominent white tissue at the proximal part of the wing (Figure 2A,B). Larval wings at a later stage are larger and marked by the invasion of tracheal tissue along the veins (Figure 2C,D). Pupal wings around 18–24 h will have prominent tracheal tissue in the wing blade (Figure 2E,F). The wings at the pupal stages are much larger and fragile than at the larval stage. Care must be taken to prevent damage to the wing tissue at this stage.

## 5. Discussion

Butterflies are becoming a model system to understand the process of color pattern formation in Biology. Over the past three decades numerous research papers have illuminated the processes involved in eyespots development in the wings of butterflies such as *Bicyclus anynana* and *Junonia coenia* [11,13,20,22,23,30,31]; color patterning and mimicry in *Heliconius* and *Papilio* butterflies [1,2,3]; and involvement of multiple signaling pathways in wing pigmentation in species belonging to the genus *Pieris*, *Junonia*, and *Colias* [4,7,9,10]. Almost all of these studies involved the process of wing dissections. The dissected wings can be used to localize proteins and gene transcripts involved in color patterning [5,12] and for more advanced techniques such as RNA, FAIRE, and ATAC sequencing [2,23]. Wing dissections, hence, are indispensable for a full understanding of the evolution and development of butterfly wing color patterns. Furthermore, experiments such as in vivo live cell imaging [28,29], used to study cellular dynamics overlap with some the wing dissection steps such as removal of cuticle and might benefit from the protocol mentioned here.

To conclude, we have provided a detailed description of the process of wing dissections in a butterfly species which we believe will be helpful for newcomers in the field to adapt to their own species.

## 6. Reagents Setup

### Preparation of 10 × PBS Buffer

1. In a 1 L beaker, add 700 mL MilliQ water and reagents mentioned in Table 1:
2. Transfer the content to a 1 L measuring cylinder. Raise the volume to one liter using MilliQ water.
3. Mix the solution and transfer the content to a 1 L glass bottle.
4. Autoclave the solution at 121 °C for 20 min and store the content at room temperature.

## Figures and Tables

**Table 1:** Reagents for 10 × PBS (Phosphate Buffer Saline) buffer preparation.

Reagents	Weight/Volume
NaCl	81.8 g
KH_2_PO_4_	5.28 g
K_2_HPO_4_	10.68 g

Note: To prepare 1 × PBS, add 10 mL of 10 × PBS buffer and 90 mL of MilliQ water.
